# Serum uric acid level as a prognostic biomarker in critically ill patients with sepsis-associated acute kidney injury: A retrospective single-center study

**DOI:** 10.1371/journal.pone.0321576

**Published:** 2025-05-07

**Authors:** Zhenkui Hu, Chao Song, Jinhui Zhang

**Affiliations:** 1 Department of Emergency Medicine, The Affiliated Hospital, Jiangsu University, Zhenjiang, Jiangsu, China; 2 Department of Critical Care Medicine, The Affiliated Hospital, Jiangsu University, Zhenjiang, Jiangsu, China; Istituto Di Ricerche Farmacologiche Mario Negri, ITALY

## Abstract

**Background:**

The predictive value of serum uric acid (SUA) for clinical outcomes in patients with sepsis-associated acute kidney injury (SA-AKI) remained unclear. Therefore, this study aimed to evaluate the clinical significance of SUA in predicting all-cause mortality among critically ill patients diagnosed with SA-AKI.

**Methods:**

This retrospective study examined 483 patients with SA-AKI at the Affiliated Hospital of Jiangsu University from January 2015 to July 2023. The primary outcome evaluated in this study was in hospital all-cause mortality. To assess the prognostic value of SUA, we employed receiver operating characteristic curves, restricted cubic splines, Cox regression models, and Kaplan-Meier survival analysis.

**Results:**

The hospital mortality and intensive care unit (ICU) mortality reached 43.3% and 42.2%, respectively. Kaplan-Meier analysis showed that the risk of 30-day mortality (log-rank test, P < 0.001) and 60-day mortality (log-rank test, P = 0.001) was significantly higher in patients with hyperuricemia (HUA). Multivariate Cox proportional hazards analysis showed that elevated SUA levels were significantly related to all-cause mortality. After accounting for potential confounding factors, patients with HUA maintained a significant correlation with both hospital mortality [HR (95%CI): 1.462 (1.094-1.952); P = 0.010] and ICU mortality [HR (95%CI): 1.474 (1.096-1.983); P = 0.010]. Further examination using restricted cubic splines revealed a progressively increasing risk of all-cause mortality with rising SUA levels.

**Conclusions:**

Higher levels of SUA were associated with an increased risk of both hospital mortality and ICU mortality in critically ill patients with SA-AKI. These findings suggested that SUA may serve as an independent prognostic marker for these patients.

## Introduction

Acute kidney injury (AKI) is characterized by a sudden and severe decline in kidney function due to various factors. Its incidence rate is high, affecting approximately 10%–25% of hospitalized patients and up to 16%–67% in intensive care units (ICUs) [1,2]. When AKI progresses to a severe stage, it becomes challenging to manage clinically, with a mortality rate remaining high at 50%–75% [3]. Among the leading causes of AKI in ICUs, such as surgery, nephrotoxic medications, trauma, and infections, sepsis accounts for 45% to 70% of cases and is often associated with a mortality rate exceeding 50% [4]. Sepsis-associated acute kidney injury (SA-AKI) refers to the onset of AKI within 7 days following the occurrence of sepsis[4]. SA-AKI patients experience a significantly increased mortality rate (2–3 times higher compared to non-SA-AKI patients), ranging from 53.3% to 74.5% versus 21.3% to 28.4% [5–8]. Furthermore, survivors often face persistent renal dysfunction and require long-term dialysis, imposing a substantial financial burden [9]. Thus, identifying the associated risk factors that impact the prognosis of SA-AKI can provide valuable insights for improved clinical diagnosis, treatment, and assessment of this condition.

Serum uric acid (SUA), as the final product of purine metabolism, is eliminated from the body through the kidneys [10]. Studies conducted on both animals and humans had revealed its inhibitory effects on the proliferation and migration of endothelial cells, ultimately leading to endothelial dysfunction and apoptosis [11]. Uric acid hindered the production of nitric oxide within endothelial cells by suppressing the activity of renal nitric oxide synthase [12]. Consequently, this could instigate renal vasoconstriction by stimulating the renin-angiotensin system [12]. Moreover, heightened level of the SUA can prompt the expression of pro-inflammatory cytokines, such as TNF-α, and local chemokines, including monocyte chemoattractant protein 1, within the kidneys. Notably, hyperuricemia activateed NADPH oxidase, thereby initiating oxidative damage in proximal tubular cells [13–15]. These alterations contributed to the initiation and escalation of inflammatory responses, enhancing cellular interactions of the immune system, eventually inducing and aggravating the progression of SA-AKI [16,17]. Meanwhile, it had been found that hyperuricemia (HUA) was associated with the prognosis of many diseases. A study had indicated a significant statistical association between the SUA level and all-cause mortality in individuals with type 2 diabetes [18]. Recent research suggested that the level of SUA could serve as a prognostic marker for hospital mortality in patients with ARDS [19]. Several reports had also linked HUA to predicting one-year mortality in acute heart failure patients, adverse outcomes in patients with acute myocardial infarction, and an increased risk of in-hospital mortality in patients with AKI [20–22]. Additionally, a study found that elevated SUA level in sepsis patients were associated with a higher risk of 90-day all-cause mortality and the incidence of AKI [23]. However, the relationship between the SUA level and adverse outcomes of SA-AKI had not been investigated. Therefore, the objective of this study was to further evaluate the predictive value of the SUA level on the prognosis of SA-AKI patients.

## Methods

### Patients and participants

In this single-center retrospective study, we reviewed the electronic medical records of consecutive adult patients with a first episode of sepsis in ICU of Affiliated Hospital of Jiangsu University from January 2015 to July 2023. The study adhered to specific criteria for including and excluding patients. Inclusion criteria involved patients who were diagnosed with sepsis according to the sepsis 3.0 criteria [24]. Additionally, sepsis patients who developed AKI within the first week of their ICU admission were included. For patients who had multiple hospital admissions, only the initial admission was considered for this study. Conversely, exclusion criteria for this study were as follows: patients under 18 years old; patients with an ICU stay less than 24 hours; AKI occurred before current ICU admission; AKI was supposed to be secondary to causes other than sepsis; patients with chronic kidney disease or hepatic cirrhosis or gout or tumor lysis; patients with missing data on SUA levels on the first day of ICU admission were excluded from the study. Additionally, any patients with variables missing more than 20% of their data were also excluded. For variables with missing data of less than 20%, we employed multiple imputation using a random forest algorithm. The research protocol obtained approval from the ethics committee of the Affiliated Hospital of Jiangsu University (Approval No. KY2023K1007). The requirement for informed consent was waived because of its retrospective design. The study was conducted according to the Strengthening the Reporting of Observational Studies in Epidemiology (STROBE) reporting guideline.

### Clinical assessment

The clinical variables utilized in this research were acquired from the electronic medical records. These variables can be classified into the following categories: Demographic characteristics: This encompassed age, gender, body mass index (BMI), and smoking status. Comorbidities: The comorbid conditions included hypertension, diabetes, coronary artery disease, chronic obstructive pulmonary disease (COPD), and cerebral infarction. Infection pathogens: This category involved the types of infection pathogens, such as gram-positive bacteria, gram-negative bacteria, fungi, and viruses. Infection locations: This included infection locations such as multisite infection, lower respiratory infection, gastrointestinal infection, intra-abdominal infection, genitourinary tract infection, bacteremia, and skin and soft tissue infection. Categorize: This encompassed early SA-AKI and late SA-AKI, as well as prerenal SA-AKI and intrarenal SA-AKI. Laboratory data: Laboratory measurements taken within 24 hours after ICU admission consisted of white blood cell (WBC), neutrophil (Neu), lymphocyte (Lym), platelet (PLT), C-reactive protein (CRP), total bilirubin (TBil), alanine transaminase (ALT), aspartate aminotransferase (AST), albumin (Alb), glucose, creatinine, blood urea nitrogen (BUN), SUA, D-dimer, potassium, and lactate. Severity scores: The severity of the patients’ conditions was assessed using two scoring systems, namely the Acute Physiology and Chronic Health Evaluation II (APACHE II) score and the Sequential Organ Failure Assessment (SOFA) score. Treatments: The treatments documented in this study included continuous renal replacement therapy (CRRT) (Emergency dialysis following KDIGO AKI stage 3), vasoactive drugs, and invasive ventilation.

### Identification of SA-AKI patients

Sepsis was identified as lifethreatening organ dysfunction caused by a dysregulated host response to infection, with organ dysfunction identified as an acute change in total SOFA score ≥  2 points. In this study, the SOFA score was calculated using the relevant indicators when the patient was admitted to the ICU. Each patient was diagnosed by an experienced ICU physician based on the Sepsis 3.0 diagnostic criteria [24]. AKI was defined by the 2012 Kidney Disease: Improving Global Outcomes Clinical Practice Guidelines (KDIGO) [25] as an elevated in serum creatinine of 0.3 mg/dL within 48 hours or a raise of at least 1.5 times the baseline level in the preceding seven days, or a decrease in urine output (UO) to less than 0.5 ml/kg/h for more than 6 hours. We excluded the UO criteria because most inpatients lacked urine volume records. The first serum creatinine value measured after ICU admission was used as the baseline serum creatinine concentration. Early SA-AKI in sepsis patients was defined as AKI occurring within 2d of ICU admission and late SA-AKI in sepsis patients was defined as AKI occurring within 2d-7d of ICU admission [26]. We defined renal function recovery as a serum creatinine value below 150% of baseline and no need for renal replacement therapy at discharge [27]. The baseline estimated glomerular filtration rate (eGFR) was calculated according to the Modification of Diet in Renal Disease study equation modified by the Chinese coefficient [28].

### Definition of hyperuricemia

Hyperuricemia (HUA) was defined as a SUA level exceeding 360.0 μmol/L in females or 420.0 μmol/L in males [29]. Patients were divided into HUA and Non-HUA groups according to the first SUA value within 24 h after the ICU admission.

### Outcomes

The primary outcome of this study was in hospital all-cause mortality. Of these, hospital death included ICU death and general ward inpatient death. The secondary outcomes included AKI recovery as well as ICU and hospital length of stay (LOS).

### Statistical analysis

The PASS software was used to calculate the test’s effectiveness. A significance level (α) of 0.05 was set, with a total sample size of 483, resulting in a power of 93.6% to analyze the relationship between SUA and patient outcomes. In this study, we presented continuous variables as medians [interquartile ranges (IQRs)] or means ±  standard deviations (SDs) and compared them using analysis of variance. Categorical variables were expressed as numbers and percentages. For comparison between different groups, either the chi-square test or Fisher’s exact test was used, as appropriate. The predictive value of the SUA level in relation to hospital mortality and ICU mortality was assessed using the area under the receiver operating characteristic curve (AUROC). We performed log-rank tests and Kaplan-Meier survival analyses to explore differences in all-cause mortality among the different groups. Furthermore, we employed multivariate Cox proportional hazards models to investigate the impact of the SUA level on all-cause mortality. Hazard ratios (HRs) with 95% confidence intervals (CIs) were reported as the results after adjusting for covariates under three different models. Model 1 did not incorporate any covariates, while Model 2 adjusted for age, gender, BMI, smoking, hypertension, diabetes, coronary artery disease, WBC, and Neu. Model 3 further adjusted for Lym, PLT, CRP, Alb, D-dimer, lactate, APACHE II score, and SOFA score. Additionally, we analyzed the non-linear relationship between SUA and hospital mortality and ICU mortality using a restricted cubic spline regression model with three knots. Subgroup analyses were performed based on age (≤65 or > 65), gender (male or female), smoking (yes or no), hypertension (yes or no), diabetes (yes or no), and lactate level (≤2.0 or > 2) to investigate potential interactions. The statistical analyses were conducted using IBM SPSS 26.0, GraphPad Prism 10.0, and R version 4.2.0 software. A two-sided P-value of less than 0.05 was considered statistically significant.

## Result

A total of 483 patients diagnosed with SA-AKI were included in the study based on the specified inclusion and exclusion criteria. The median age of the participants was 76 (67-84) years, with 296 (61.3%) being female, and the median BMI was 22.49 (19.98-25.39) kg/m^2^. Hypertension was the most prevalent comorbidity, affecting 54.5% of the patients, followed by diabetes (31.3%), cerebral infarction (14.5%), coronary artery disease (13.3%), and COPD (6.8%). Among the participants, 72 (14.9%) required CRRT, and 386 (79.9%) received vasoactive drugs. Additionally, 327 (67.7%) required invasive ventilation. The median LOS in the ICU was 7 (4-12) days, and the median LOS in the hospital was 16 (9-25) days. The hospital mortality and ICU mortality rates were 44.3% and 42.2%, respectively. Further baseline characteristics can be found in Table 1.

**Table 1:** Baseline demographic and clinical characteristics by different SUA levels in S-AKI patients.

Variables	Overall	Non-HUA group	HUA group	P-value
N	483	239	244	–
Age, years	76 (67-84)	76 (66-85)	77 (67-84)	0.953
Female, n (%)	296 (61.3)	78 (32.6)	109 (44.7)	0.007
BMI, kg/m^2^	22.49 (19.98-25.39)	22.22 (19.59-24.86)	22.74 (20.43-25.95)	0.098
Smoking, n (%)	84 (17.4)	45 (18.8)	39 (16.0)	0.410
**Comorbidities, n (%)**				
Hypertension	263 (54.5)	123 (51.5)	140 (57.4)	0.192
Diabetes	151 (31.3)	69 (28.9)	82 (33.6)	0.262
Coronary artery disease	64 (13.3)	26 (10.9)	38 (15.6)	0.128
COPD	33 (6.8)	19 (7.9)	14 (5.7)	0.335
Cerebral infarction	70 (14.5)	38 (15.9)	32 (13.1)	0.385
**Infection pathogens, n (%)**				
Gram-positive bacteria	62 (12.8)	31 (13.0)	31 (12.7)	0.930
Gram-negative bacteria	171 (35.4)	85 (35.6)	86 (35.2)	0.942
Fungus	49 (10.4)	28 (11.7)	21 (8.6)	0.258
Virus	17 (3.5)	10 (4.2)	7 (2.9)	0.433
**Infection sites, n (%)**				
Multisite Infection	73 (15.1)	30 (12.6)	43 (17.6)	0.120
Lower respiratory infection	136 (28.2)	62 (25.9)	74 (30.3)	0.284
Gastrointestinal infection	9 (1.9)	4 (1.7)	5 (2.0)	0.760
Intra-abdominal infection	174 (29.5)	102 (42.7)	72 (29.5)	0.003
Genitourinary tract infection	50 (10.4)	27 (11.3)	23 (9.4)	0.500
Bacteremia	8 (1.7)	2 (0.8)	6 (2.5)	0.163
Skin and soff tissue infection	33 (6.8)	12 (5.0)	21 (8.6)	0.118
**Categorize, n (%)**				
Early SA-AKI	308 (63.8)	160 (66.9)	148 (60.7)	0.150
Late SA-AKI	175 (36.2)	79 (33.1)	96 (39.3)	
Prerenal SA-AKI	210 (43.5)	99 (41.4)	111 (45.5)	0.367
Intrarenal SA-AKI	273 (56.5)	140 (58.6)	133 (54.5)	
**Laboratory tests**				
WBC * 10^9^/L	12.8 (7.9-18.6)	11.4 (6.7-16.8)	14.2 (9.3-21.1)	<0.001
Neu * 10^9^/L	11.8 (6.8-17.1)	10.4 (5.8-15.9)	13.1 (8.0-18.9)	<0.001
Lym * 10^9^/L	0.5 (0.3-0.8)	0.5 (0.3-0.8)	0.5 (0.3-0.9)	0.235
Hb, g/dL	112 (93-129)	109 (93-125)	115 (93-133)	0.075
PLT * 10^9^/L	129 (78-193)	133 (82-197)	127 (71-190)	0.353
CRP, mg/L	128.0 (61.0-182.2)	113.0 (48.8-165.9)	136.5 (76.8-201.0)	0.002
Tbil, μmol/L	20.0 (12.1-34.4)	19.7 (11.9-34.2)	21.3 (12.4-34.5)	0.589
ALT, U/L	36.4 (23.0-75.0)	35.0 (22.0-62.0)	39.0 (23.0-87.8)	0.092
AST, U/L	50.0 (26.0-125.0)	45.0 (24.0-101.0)	56.0 (28.7-194.0)	0.010
Alb, g/L	27.1 (23.2-32.5)	26.1 (22.9-30.2)	29.1 (23.6-34.9)	<0.001
Glucose, mmol/L	8.8 (6.7-13.2)	8.5 (6.7-12.2)	9.2 (6.8-14.2)	0.022
Creatinine, μmol/L	159.5 (126.3-239.4)	136.1 (120.7-179.8)	192.6 (142.4-319.1)	<0.001
eGFR, (ml/min/1.73m^2^)	29.04 (18.43-37.10)	32.42 (25.40-39.08)	24.04 (14.67-32.98)	<0.001
BUN, mmol/L	13.79 (9.69-20.76)	11.38 (8.43-15.79)	18.61 (11.92-24.95)	<0.001
BCR	0.076 (0.053-0.108)	0.074 (0.512-0.100)	0.078 (0.056-0.114)	0.033
SUA, μmol/L	403.1 (300.9-523.7)	300.8 (260.3-344.7)	522.6 (452.8-628.0)	<0.001
D-dimer, mg/L	3.81 (3.33-4.48)	5.6 (2.9-10.0)	6.9 (3.1-13.0)	0.123
Potassium, mmol/L	2.6 (1.8-5.1)	3.8 (3.3-4.3)	3.8 (3.4-4.8)	0.078
Lactate, mmol/L	12.8 (7.9-18.6)	2.6 (1.9-4.8)	2.7 (1.8-5.7)	0.538
**Severity scoring**				
APACHE II score	27 (21-33)	27 (21-32)	27 (21-33)	0.778
SOFA score	13 (11-15)	12 (10-14)	13 (11-15)	0.052
**Treatments**				
CRRT, n (%)	72 (14.9)	29 (12.1)	72 (14.9)	0.090
Vasoactive drug, n (%)	386 (79.9)	196 (82.0)	190 (77.9)	0.256
Invasive ventilation, n (%)	327 (67.7)	168 (70.3)	159 (65.2)	0.228
**Endpoints**				
AKI recovery	215 (44.5)	128 (53.6)	87 (35.7)	<0.001
ICU length of stay, days	7 (4-12)	6 (4-11)	7 (4-13)	0.009
Hospital length of stay, days	16 (9-25)	17 (11-25)	14 (8-23)	0.019
ICU mortality, n (%)	204 (42.2)	83 (34.7)	121 (49.6)	0.001
Hospital mortality, n (%)	214 (44.3)	88 (36.8)	126 (51.6)	0.001

Abbreviations: BMI, body mass index; COPD, chronic obstructive pulmonary disease; WBC, white blood cell; Neu, neutrophil; Lym, lymphocyte; PLT, platelet; CRP, C-reactive protein; TBil, total bilirubin; ALT, alanine transaminase; AST, aspertate aminotransferase; Alb, albumin; eGFR: estimated glomerular filtration rate; BUN, blood urea nitroge; BCR, blood urea nitrogen to creatinine ratio; SUA, serum uric acid; APACHE II, Acute Physiology and Chronic Health Evaluation II; SOFA, Sequential Organ Failure Assessment; CRRT, continuous renal replacement therapy.

### Basic clinical characteristics

In accordance with the sex-specific criteria for HUA [29], the patients were divided into two distinct groups: the Non-HUA group and the HUA group. A total of 239 patients were assigned to the Non-HUA group, whereas 244 patients were classified into the HUA group. The median value of SUA for each group was 300.8 (260.3-344.7) for the Non-HUA group and 522.6 (452.8-628.0) for the HUA group. Significant variations in several laboratory parameters were observed between the two groups, as detailed in Table 1. Specifically, the HUA group exhibited significantly elevated levels of WBC, Neu, AST, glucose, creatinine, eGFR, BUN, and BCR (P < 0.05). The HUA group also displayed an increased risk of both hospital mortality and ICU mortality compared to the Non-HUA group (P < 0.001).

**Fig 1:**
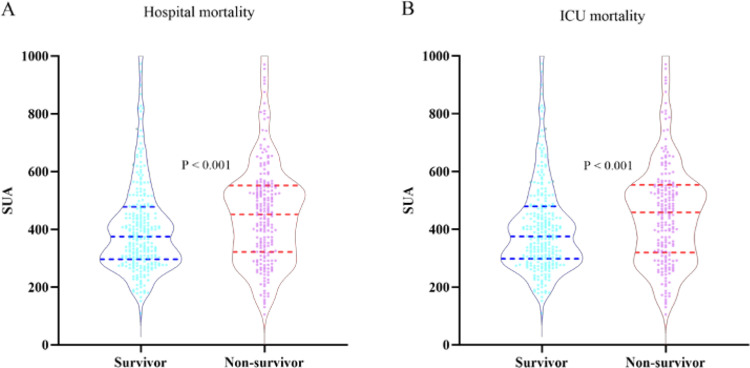
A. Boxplots of the SUA showing the distribution in the Survivor group and Non-Survivor group during hospital stay. B. Boxplots of the SUA showing the distribution in the Survivor group and Non-Survivor group during ICU stay. Abbreviations: SUA, serum uric acid; ICU, Intensive Care Unit.

Table 2 presented the differences in baseline characteristics between survivors and non-survivors during the hospital stay. The group of non-survivors were generally older and had a higher prevalence of coronary artery disease, COPD, cerebral infarction, late SA-AKI and prerenal SA-AKI. Moreover, they demonstrated higher levels of ALT, AST, glucose, creatinine, eGFR, BUN, D-dimer, and lactate, along with lower levels of Lym and Hb. The non-survivors also exhibited more severe illness scores and a greater proportion of individuals requiring CRRT, vasoactive drugs, and invasive ventilation compared to the survivor group. Notably, the SUA levels in the non-survivor group were significantly higher compared to those in the survivor group (451.8 vs. 375.0, P < 0.001). Figs 1A and 1B depicted the distribution of SUA categorized by the mortality status of all-cause hospital death and ICU death, respectively.

**Table 2:** Baseline characteristics of the Survivors and Non-Survivors groups.

Variables	Overall	Survivors group	Non-Survivors group	P-value
N	483	269	214	–
Age, years	76 (67-84)	74 (65-82)	80 (72-86)	<0.001
Male, n (%)	296 (61.3)	114 (42.4)	73 (34.1)	0.064
BMI, kg/m^2^	22.49 (19.98-25.39)	23.03 (20.43-25.71)	22.04 (19.56-24.97)	0.033
Smoking, n (%)	84 (17.4)	42 (15.6)	42 (19.6)	0.248
**Comorbidities, n (%)**				
Hypertension	263 (54.5)	144 (53.5)	119 (55.6)	0.649
Diabetes	151 (31.3)	81 (30.1)	70 (32.7)	0.541
Coronary artery disease	64 (13.3)	28 (10.4)	36 (16.8)	0.039
COPD	33 (6.8)	12 (4.5)	21 (9.8)	0.021
Cerebral infarction	70 (14.5)	27 (10.0)	43 (20.1)	0.002
**Categorize, n (%)**				
Early SA-AKI	308 (63.8)	198 (73.6)	110 (51.4)	<0.001
Late SA-AKI	175 (36.2)	71 (26.4)	104 (48.6)	
Prerenal SA-AKI	210 (43.5)	105 (39.0)	105 (49.1)	0.027
Intrarenal SA-AKI	273 (56.5)	164 (61.0)	109 (50.9)	
**Infection pathogens, n (%)**				
Gram-positive bacteria	62 (12.8)	32 (11.9)	30 (14.0)	0.488
Gram-negative bacteria	171 (35.4)	97 (36.1)	74 (34.6)	0.735
Fungus	49 (10.4)	17 (6.3)	32 (15.0)	0.002
Virus	17 (3.5)	3 (1.1)	14 (6.5)	0.001
**Infection sites, n (%)**				
Multisite Infection	73 (15.1)	40 (14.9)	33 (15.4)	0.867
Lower respiratory infection	136 (28.2)	41 (15.2)	95 (44.4)	<0.001
Gastrointestinal infection	9 (1.9)	6 (2.2)	3 (1.4)	0.504
Intra-abdominal infection	174 (29.5)	118 (43.9)	56 (26.2)	<0.001
Genitourinary tract infection	50 (10.4)	40 (14.9)	10 (4.7)	<0.001
Bacteremia	8 (1.7)	5 (1.9)	3 (1.4)	0.696
Skin and soff tissue infection	33 (6.8)	18 (6.7)	15 (7.0)	0.891
**Laboratory tests**				
WBC * 10^9^/L	12.8 (7.9-18.6)	12.4 (7.4-19.1)	13.3 (8.7-18.4)	0.359
Neu * 10^9^/L	11.8 (6.8-17.1)	11.4 (6.6-17.3)	12.2 (7.8-17.2)	0.260
Lym * 10^9^/L	0.5 (0.3-0.8)	0.5 (0.3-1.0)	0.4 (0.3-0.7)	<0.001
Hb, g/dL	112 (93-129)	116 (97-131)	106 (88-126)	0.002
PLT * 10^9^/L	129 (78-193)	133 (83-204)	121 (66-180)	0.010
CRP, mg/L	128.0 (61.0-182.2)	128.0 (60.6-182.0)	126.8 (61.3-186.7)	0.945
Tbil, μmol/L	20.0 (12.1-34.4)	19.9 (12.8-32.4)	21.0 (11.4-38.7)	0.808
ALT, U/L	36.4 (23.0-75.0)	34.0 (22.0-60.5)	40.0 (23.0-94.3)	0.040
AST, U/L	50.0 (26.0-125.0)	40.0 (24.0-87.4)	65.0 (30.8-216.6)	<0.001
Alb, g/L	27.1 (23.2-32.5)	27.6 (23.6-33.2)	26.6 (22.6-31.7)	0.051
Glucose, mmol/L	8.8 (6.7-13.2)	8.2 (6.6-12.8)	9.2 (7.1-14.1)	0.026
Creatinine, μmol/L	159.5 (126.3-239.4)	150.0 (123.8-224.0)	169.0 (131.8-282.9)	0.002
eGFR, (ml/min/1.73m^2^)	29.04 (18.43-37.10)	31.18 (20.89-39.53)	26.49 (15.72-33.75)	<0.001
BUN, mmol/L	13.79 (9.69-20.76)	12.26 (9.19-18.51)	16.78 (10.50-23.15)	<0.001
BCR	0.076 (0.053-0.108)	0.075 (0.054-0.099)	0.079 (0.052-0.119)	0.089
SUA, μmol/L	403.1 (300.9-523.7)	375.0 (296.0-478.0)	451.8 (321.9-552.0)	<0.001
D-dimer, mg/L	3.81 (3.33-4.48)	5.1 (2.5-9.8)	7.2 (3.7-13.7)	0.001
Potassium, mmol/L	2.6 (1.8-5.1)	3.8 (3.3-4.4)	3.8 (3.4-4.7)	0.091
Lactate, mmol/L	12.8 (7.9-18.6)	2.3 (1.7-4.0)	3.6 (2.1-6.6)	<0.001
**Severity scoring**				
APACHE II score	27 (21-33)	24 (20-30)	29 (25-35)	<0.001
SOFA score	13 (11-15)	12 (10-14)	14 (12-16)	<0.001
**Treatments**				
CRRT, n (%)	72 (14.9)	15 (5.6)	57 (26.6)	<0.001
Vasoactive drug, n (%)	386 (79.9)	188 (69.9)	198 (92.5)	<0.001
Invasive ventilation, n (%)	327 (67.7)	134 (49.8)	193 (90.2)	<0.001

Abbreviations: BMI, body mass index; COPD, chronic obstructive pulmonary disease; WBC, white blood cell; Neu, neutrophil; Lym, lymphocyte; PLT, platelet; CRP, C-reactive protein; TBil, total bilirubin; ALT, alanine transaminase; AST, aspertate aminotransferase; Alb, albumin; eGFR: estimated glomerular filtration rate; BUN, blood urea nitroge; BCR, blood urea nitrogen to creatinine ratio; BUN, blood urea nitroge; SUA, serum uric acid; APACHE II, Acute Physiology and Chronic Health Evaluation II; SOFA, Sequential Organ Failure Assessment; CRRT, continuous renal replacement therapy.

### Association between the all-cause mortality and SUA

**Fig 2:**
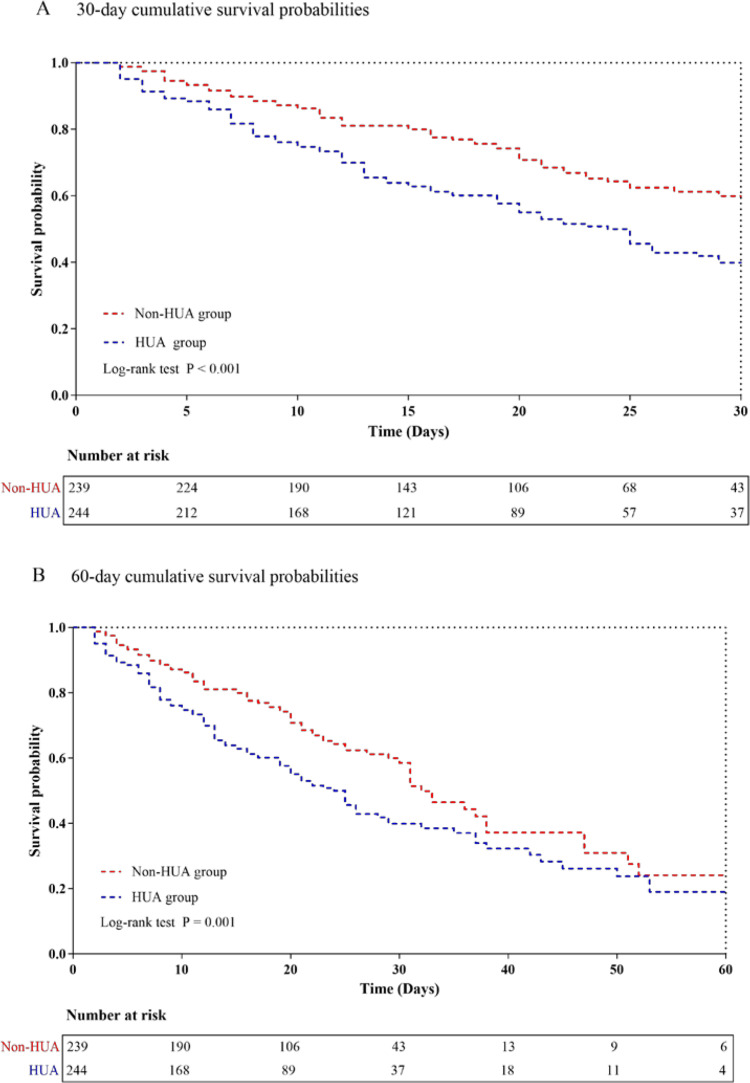
Kaplan-Meier curves showing cumulative probability of all-cause mortality according to groups at 30 days (A), and 60 days (B). Abbreviations: HUA, hyperuricemia.

To analyze the occurrence of primary outcomes in the two groups, we utilized Kaplan-Meier survival analysis (Figs 2A and 2B). The results showed that patients with HUA had a higher risk of both 30-day mortality (log-rank test, P < 0.001) and 60-day mortality (log-rank test, P = 0.001). We evaluated the clinical effectiveness of SUA using ROC analysis. However, the AUC values for SUA did not demonstrate satisfactory performance, indicating its limited predictive capability for hospital mortality (AUC: 0.596, P < 0.001) and ICU mortality (AUC: 0.596, P < 0.001) (S1 Fig).

**Fig 3:**
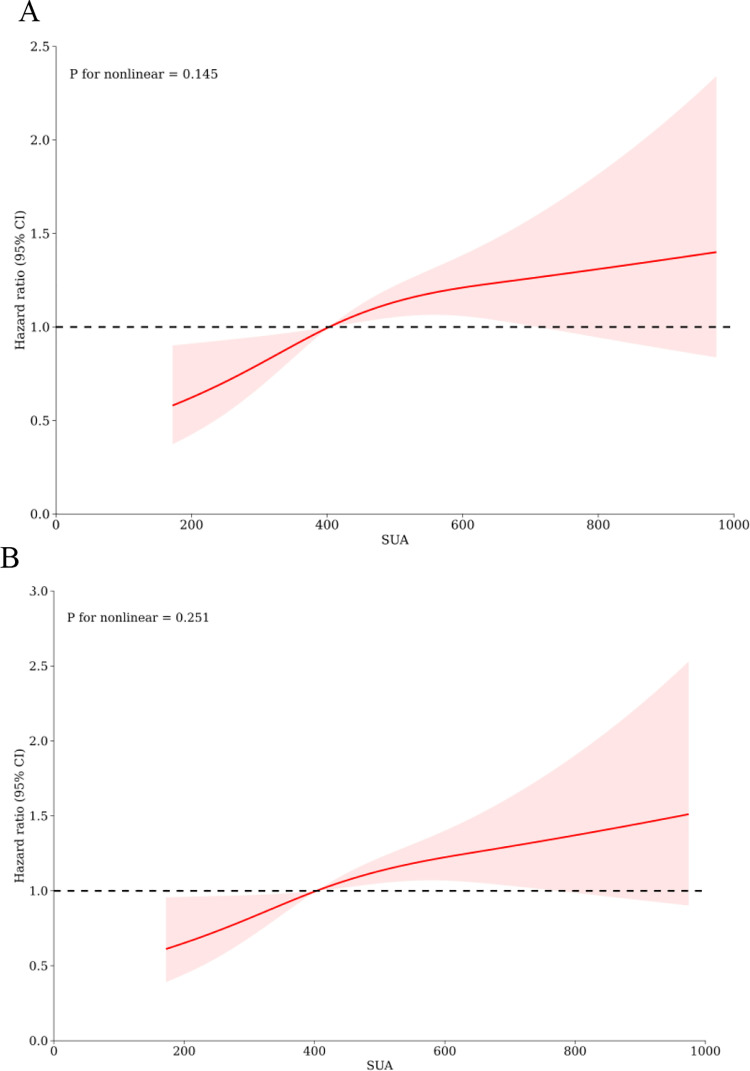
Restricted cubic spline regression analysis of SUA with in hospital all-cause mortality. Heavy central lines represent the estimated adjusted hazard ratios, with shaded ribbons denoting 95% confidence intervals. The horizontal dotted lines represent the hazard ratio of 1.0. A. Restricted cubic spline for hospital mortality. B. Restricted cubic spline for ICU mortality. Abbreviations: SUA, serum uric acid; ICU, Intensive Care Unit.

The results of the univariate Cox regression analysis, evaluating the risk of all-cause mortality in critically ill patients with SA-AKI, were presented in S1 Table. Only variables with a significance level of P < 0.05 were included, as well as factors suggested by clinicians based on their clinical experience. The influential factors identified through this analysis were age, gender, BMI, smoking, hypertension, diabetes, coronary artery disease, WBC, Neu, Lym, PLT, CRP, Alb, D-dimer, lactate, APACHE II score, and SOFA score. In the three multivariate Cox proportional hazard models, when SUA was treated as a continuous variable, it showed a significant positive correlation with hospital mortality, both when increased by 1 unit or 1 standard deviation (SD). When SUA was considered as a nominal variable, patients in the HUA group had a significantly higher risk of hospital death compared to those in the non-HUA group, as demonstrated in the three established Cox proportional hazards models: unadjusted model [HR (95%CI): 1.566 (1.192-2.058); P = 0.001], partly adjusted model [HR (95%CI): 1.593 (1.199-2.117); P = 0.001], and fully adjusted model [HR (95%CI): 1.462 (1.094-1.952); P = 0.010] (Table 3). Similar results were observed in the multivariate Cox proportional risk analysis of SUA and ICU mortality, as shown in Table 3. Additionally, the restricted cubic splines regression model depicted a linear increase in the risk of both hospital mortality (P for non-linearity = 0.145) and ICU mortality (P for non-linearity = 0.251) with rising SUA levels (Figs 3A and 3B).

**Table 3:** Multivariate Cox proportional hazards models for hospital mortality and ICU mortality based on the SUA level.

Variables	Model 1		Model 2		Model 3	
	**HR (95%CI)**	**P**	**HR (95%CI)**	**P**	**HR (95%CI)**	**P**
**Hospital mortality**						
Per unit increase	1.001 (1.000-1.002)	0.002	1.001 (1.000-1.002)	0.002	1.001 (1.000-1.002)	0.024
Per SD increase	1.215 (1.077-1.371)	0.002	1.219 (1.076-1.380)	0.002	1.167 (1.021-1.334)	0.024
Non-HUA	Ref		Ref		Ref	
HUA	1.566 (1.192-2.058)	0.001	1.593 (1.199-2.117)	0.001	1.462 (1.094-1.952)	0.010
**ICU mortality**						
Per unit increase	1.001 (1.000-1.002)	0.002	1.001 (1.000-1.002)	0.002	1.001 (1.000-1.002)	0.027
Per SD increase	1.206 (1.073-1.357)	0.002	1.229 (1.082-1.397)	0.002	1.168 (1.018-1.339)	0.027
Non-HUA	Ref		Ref		Ref	
HUA	1.580 (1.195-2.091)	0.001	1.615 (1.207-2.161)	0.001	1.474 (1.096-1.983)	0.010

Note: Model 1: Unadjusted model.

Model 2: Adjusted for age, gender, BMI, smoking, hypertension, diabetes, coronary artery disease, WBC, and Neu.

Model 3: Model 2, with additional adjustments for Lym, PLT, CRP, Alb, D-dimer, lactate, APACHE II score, and SOFA score.

Abbreviations: HUA, hyperuricemia; BMI, body mass index; WBC, white blood cell; Neu, neutrophil; Lym, lymphocyte; PLT, platelet; CRP, C-reactive protein; Alb, albumin; SUA, serum uric acid; APACHE II, Acute Physiology and Chronic Health Evaluation II; SOFA, Sequential Organ Failure Assessment; CRRT, continuous renal replacement therapy.

### Subgroup analysis

The study further examined the risk assessment significance of HUA for primary outcomes across various subgroups of participants. These subgroups included age (≤65 or > 65), gender (male or female), smoking (yes or no), hypertension (yes or no), diabetes (yes or no), and lactate levels (≤2.0 or > 2.0) (Table 4 and Table 5). Subgroup analysis revealed a significant association between HUA and an elevated risk of hospital mortality within the following subgroups: patients aged > 65 years [HR (95%CI): 1.535 (1.139-2.068); P = 0.005], male patients [HR (95%CI): 1.406 (1.009-1.958); P = 0.044], female patients [HR (95%CI): 1.997 (1.194-3.341); P = 0.008], patients without smoking [HR (95%CI): 1.813 (1.332-2.468); P < 0.001], patients with hypertension [HR (95%CI): 1.701 (1.174-2.464); P = 0.005], patients without hypertension [HR (95%CI): 1.514 (1.003-2.285); P = 0.049], patients without diabetes [HR (95%CI): 1.788 (1.281-2.494); P = 0.001], and patients with lactate level > 2.0 mmol/L [HR (95%CI): 1.725 (1.261-2.358); P = 0.001]. Notably, the prognostic significance of HUA seemed to be more pronounced among non-smoking patients [HR (95%CI) without smoking 1.813 (1.332-2.468) versus with smoking 0.848 (0.458-1.570), p-value for interaction = 0.027]. Similar findings were observed in the subgroup analyses investigating the relationship between HUA and ICU mortality (Table 5).

**Table 4:** Subgroup analysis regarding the influence of different SUA levels in the hospital mortality.

Subgroups	No. hospital mortality/No. patients	HR (95% CI)	P-value	P for interaction
Age				0.631
>65	178/377	1.535 (1.139-2.068)	0.005	
≤65	36/106	1.790 (0.903-3.548)	0.095	
Gender				0.689
Male	141/296	1.406 (1.009-1.958)	0.044	
Female	73/187	1.997 (1.194-3.341)	0.008	
Smoking				0.027
Yes	42/84	0.848 (0.458-1.570)	0.600	
No	172/399	1.813 (1.332-2.468)	<0.001	
Hypertension				0.625
Yes	119/263	1.701 (1.174-2.464)	0.005	
No	95/220	1.514 (1.003-2.285)	0.049	
Diabetes				0.139
Yes	70/151	1.138 (0.705-1.837)	0.596	
No	144/332	1.788 (1.281-2.494)	0.001	
Lactate				0.182
>2.0	167/337	1.725 (1.261-2.358)	0.001	
≤2.0	47/146	1.117 (0.629-1.983)	0.705	

Abbreviations: SUA, serum uric acid.

**Table 5:** Subgroup analysis regarding the influence of different SUA levels in the ICU mortality.

Subgroups	No. ICU mortality/No. patients	HR (95% CI)	P-value	P for interaction
Age				0.808
>65	169/377	1.570 (1.156-2.133)	0.004	
≤65	35/106	1.709 (0.857-3.407)	0.128	
Gender				0.362
Male	136/296	1.462 (1.043-2.048)	0.028	
Female	68/187	1.942 (1.143-3.299)	0.014	
Smoking				0.024
Yes	42/84	0.848 (0.458-1.570)	0.600	
No	162/399	1.846 (1.343-2.538)	<0.001	
Hypertension				0.387
Yes	113/263	1.813 (1.236-2.660)	0.002	
No	91/220	1.429 (0.941-2.169)	0.094	
Diabetes				0.237
Yes	68/151	1.223 (0.750-1.993)	0.420	
No	136/332	1.753 (1.246-2.468)	0.001	
Lactate				0.150
>2.0	162/337	1.744 (1.270-2.395)	0.001	
≤2.0	42/146	1.067 (0.581-1.958)	0.835	

Abbreviations: SUA, serum uric acid; ICU, Intensive Care Unit.

## Discussion

To the best of our knowledge, this study was the first to explore the relationship between SUA and all-cause mortality in SA-AKI patients. Our primary finding indicated that increased SUA was a strong independent predictor of both hospital mortality and ICU mortality in SA-AKI patients. This association remained after adjustment for a wide range of clinical and laboratory variables. Thus, the SUA might be a promising decision-making tool for clinicians and could serve as an independent risk factor in SA-AKI patients.

It was well known that the correlation between SUA and AKI was first discovered in studies on tumor lysis syndrome, where the deposition of uric acid crystals in the kidneys caused a decrease in glomerular filtration rate (GFR) and induced local kidney inflammation through a crystal-dependent mechanism [30]. However, numerous studies had shown that SUA can also participate in the occurrence and development of AKI through non-crystal-dependent mechanisms. Animal models had found that mild hyperuricemia led to enhanced renal vasoconstriction, with GFR and renal blood flow decreasing by 50% [31]. Monocytes from healthy volunteers, when exposed to uric acid infusion for 24 hours, were found to selectively promote the production of the pro-inflammatory cytokine IL-1β while inhibiting the synthesis of the natural inhibitor IL-1 receptor antagonist (IL-1Ra), shifting the balance of IL-1/IL-1Ra towards a pro-inflammatory phenotype and further exacerbating kidney injury [32]. Xiao et al. also found that soluble uric acid can enhance the expression of the NALP3 inflammatory complex in renal tubular epithelial cells via the Toll-4R-dependent pathway, activating the innate immune response in human renal tubular epithelial cells and participating in kidney injury [33]. In addition, SUA can also contribute to kidney injury by activating the renin-angiotensin system (RAS), stimulating vascular smooth muscle cell proliferation, and increasing oxidative stress [34]. Although the physiological mechanisms by which SUA led to AKI were not fully understood, current research suggested that SUA may be involved in the occurrence and development of AKI through inflammation and oxidative stress [35,36]. Inflammation and oxidative stress were important pathological and physiological processes in sepsis [37], thus SUA level may reflect the inflammatory and oxidative stress in SA-AKI patients.

In the past, SUA was considered an inert waste product of purine metabolism [38]. However, research in recent years had found that the SUA exhibited broad biological activity and increased SUA was associated with various disease risk factors. For instance, in a retrospective study done in China involving 1,149 COVID-19 patients found that SUA levels over 6.7 mg/dL were associated with higher levels of the inflammatory markers such as tumor necrosis factor-α (TNF-a), Interleukin-6 (IL-6), and ferritin [39]. A study at a hospital in Wuhan involving 174 COVID-19 patients demonstrated that SUA served as an independent predictor of AKI, with moderate accuracy for predicting AKI (AUC 0.71) [40]. A recent study conducted in 2022, which involved 834 patients with COVID-19, it was found that higher SUA levels were independently associated with both AKI and in-hospital mortality in a dose-dependent manner [41]. Another study by Jiang et al., which examined 634 patients with sepsis, reported that HUA was an independent risk factor for AKI in sepsis patients and hyperuricemia may be involved in the pathogenesis of sepsis-related AKI [42]. Akbar et al. conducted a prospective cohort study of 144 sepsis patients in the ICU, finding that elevated SUA levels were correlated with poor prognoses in sepsis and increased risk of AKI [19]. Another prospective cohort study identified HUA as a potential indicator of 90-day mortality in sepsis patients [23]. Additionally, two studies demonstrated that SUA level could predict the severity and prognosis of sepsis patients [19,43]. Li et al. also discovered that elevated SUA level were associated with an increased risk of in-hospital mortality in patients with AKI [22]. However, there was currently no available research investigating the relationship between SUA and clinical outcomes in patients with SA-AKI. In our present study, we observed that higher levels of SUA in SA-AKI patients were associated with a higher risk of hospital mortality and ICU mortality. These findings suggested that early elevation of SUA level could potentially serve as a prognostic factor for all-cause mortality in SA-AKI patients.

In addition, this study further analyzed the risk stratification of various subgroups. Our subgroup analysis discovered that the prognostic value of HUA in terms of mortality in hospital and ICU remained consistent for both male and female patients. However, no association was found between HUA and overall mortality during hospitalization among patients with hypertension and diabetes included in the study. This observation could potentially be explained by reverse causality, where patients who had already been diagnosed with these comorbidities were more likely to have received appropriate treatment or adopted healthier lifestyle habits. Consequently, their prognosis might have improved despite being at a higher risk for overall mortality. Furthermore, the current study revealed that the predictive value of HUA for overall mortality appeared to be more pronounced in non-smoking patients. This suggested that smoking might have a substantial influence on the predictive accuracy of HUA for overall mortality. The inconsistency could be attributed to the fact that smoking led to the generation of peroxides, which resulted in the consumption of uric acid and subsequently reduced the mortality rate in patients [44]. Previous studies had demonstrated that smokers had significantly lower levels of SUA compared to non-smokers [45]. Although research indicated that smoking can lower SUA levels, it can also induce or worsen conditions such as hypertension, coronary heart disease, and respiratory system disorders [46]. Another noteworthy finding from this study was the correlation between HUA and age, as well as its effect on overall mortality, particularly among elderly patients. This suggested that healthcare providers should pay more attention to elderly patients due to their increased likelihood of having concurrent medical conditions. Furthermore, our study emphasized the importance of equally considering younger patients, as they also faced a risk of mortality. Importantly, our study established a significant linear relationship between SUA levels and in hospital all-cause mortality, indicating that SUA could serve as a convenient indicator for identifying critically ill patients with SA-AKI who were at a heightened risk of mortality. Therefore, improving the regulation of SUA levels may potentially contribute to mitigating unfavorable clinical outcomes in critically ill patients with SA-AKI.

Is there any clear evidence supporting that lowering SUA level can improve the prognosis of SA-AKI? Currently, there is no definitive evidence to support this claim. Febuxostat, a selective and potent inhibitor of xanthine oxidase (XO), can alleviate AKI, which may be related to antioxidant stress, anti-inflammation, and antiendoplasmic reticulum stress and reducing uric acid production [47–49]. For instance, Ramos and colleagues demonstrated that febuxostat improved renal function in experimental SA-AKI animals induced by lipopolysaccharide (LPS), potentially through its antioxidant, anti-inflammatory, and antiapoptotic effects [50]. Similarly, Ibrahim et al. confirmed the protective effects of febuxostat against liver and kidney injuries in sepsis following cecal ligation, attributing this protection to its antioxidant, anti-inflammatory, and anti-apoptotic properties, as well as the attenuation of the c-Jun N-terminal kinase signaling pathway [51]. Wang et al. found that febuxostat as an XO-specific inhibitor showed antioxidant stress and anti-inflammatory effects and weakened the local hypoxia of renal tubular epithelial cells by inhibiting XO activity, thus alleviating SA-AKI. The study also demonstrated that pretreatment with febuxostat in vivo significantly improved the prognosis of SA-AKI mice by reducing the levels of BUN, serum creatinine, TNF-α, IL-6, and interleukin-1β (IL-1β) in peripheral blood and by improving histological damage, reducing kidney tubular cell apoptosis and reactive oxygen species (ROS) production, and inhibiting infiltration of neutrophils and macrophages in the kidneys [52]. Moreover, a meta-analysis of uric acid-lowering treatment in asymptomatic HUA revealed that allopurinol had a renal protective effect without an increased risk of safety events [53]. In addition to traditional uric acid-lowering drugs, animal experiments had suggested that mesenchymal stem cells can effectively attenuate uric acid-induced kidney injury through paracrine mechanisms, offering a potential therapeutic approach for uric acid-related renal damage [54]. Based on these findings, it was hypothesized that early intervention to lower SUA levels might improve the prognosis of SA-AKI. Based on these findings, it was hypothesized that early intervention to lower SUA levels might improve the prognosis of SA-AKI.

There are several limitations to our study that need to be considered. Firstly, it was important to note that this was a retrospective study conducted at a single center. Additionally, some patients had missing data for serum creatinine or SUA levels, as well as incomplete information in other areas, which could introduce selection bias. These exclusions may limit the overall representativeness of the study population. Secondly, the study did not include an analysis of long-term mortality as the researchers lacked access to mortality rates after discharge. Therefore, the impact of SUA on long-term outcomes could not be assessed and future studies should aim to explore this aspect. Thirdly, our study focused solely on the impact of hyperuricemia on the prognosis of patients with SA-AKI. We had not specifically investigated how the phenotypes of hyperuricemia (such as renal underexcretion or renal overload) affected the prognostic outcomes of different phenotypes of SA-AKI (for instance, oliguric vs. non-oliguric, prerenal vs. renal). In the future, we aim to conduct a more detailed analysis of these prognostic results. Additionally, certain unmeasured confounders, such as metabolic diseases, and prehospital intervention, volume status, nephrotoxins drugs, diuretics were not accounted for in the analysis and could have an impact on the association between SUA levels and outcomes. Furthermore, our study only examined SUA level within 24 hours of ICU admission in patients with SA-AKI and failed to evaluate the dynamic effect of SUA level. Future studies should explore the predictive value of SUA levels for SA-AKI by analyzing these dynamic changes. Lastly, the underlying mechanism linking high SUA level and prognosis remained unclear. Further research are needed to better understand the mechanisms involved. In order to validate the results and gain a deeper understanding of the mechanisms underlying these findings, it would be beneficial to conduct large-scale prospective studies involving multiple centers.

## Conclusion

In summary, our results extended the utility of the SUA to critically ill patients with SA-AKI and demonstrated that the SUA could be used as a potential index for risk stratification of hospital mortality and ICU mortality among these patients. However, further research is needed to validate our findings and gain a deeper understanding of the mechanisms through which SUA influences SA-AKI patients.

## Supporting information

S1 Fig The predictive value of SUA for all-cause mortality by ROC analysis. (DOCX)

S1 Table Cox proportional hazards regression of the factors influencing all-cause death of the study population. (DOCX)
