# Supplementary material for: Serum uric acid level as a prognostic biomarker in critically ill patients with sepsis-associated acute kidney injury: A retrospective single-center study
Source: PLoS One. 2025 May 7;20(5):e0321576. doi: 10.1371/journal.pone.0321576 (PMC12057918; doi:10.1371/journal.pone.0321576)
Supplement: S1 Fig — (DOCX) [file pone.0321576.s001.docx]

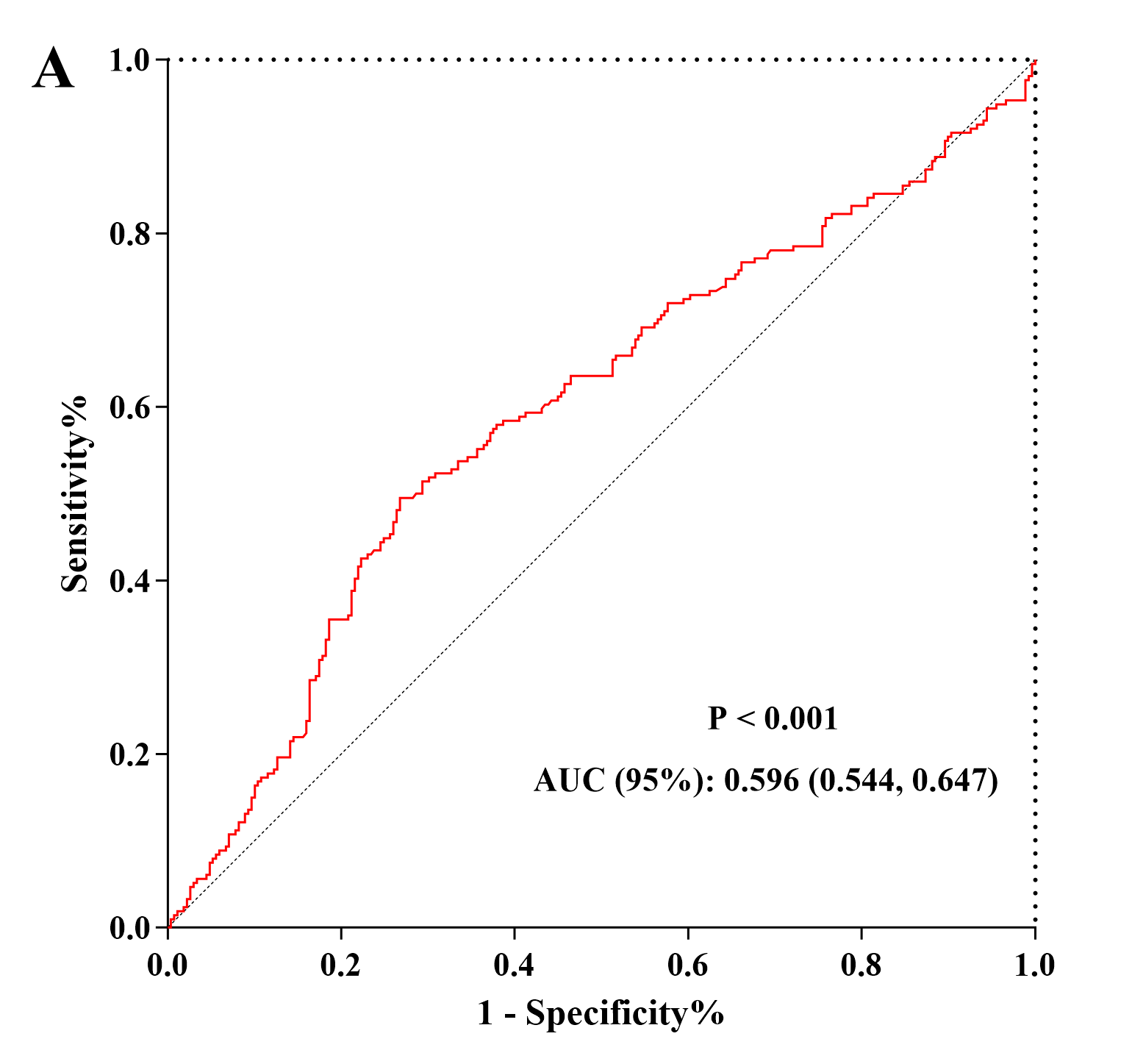

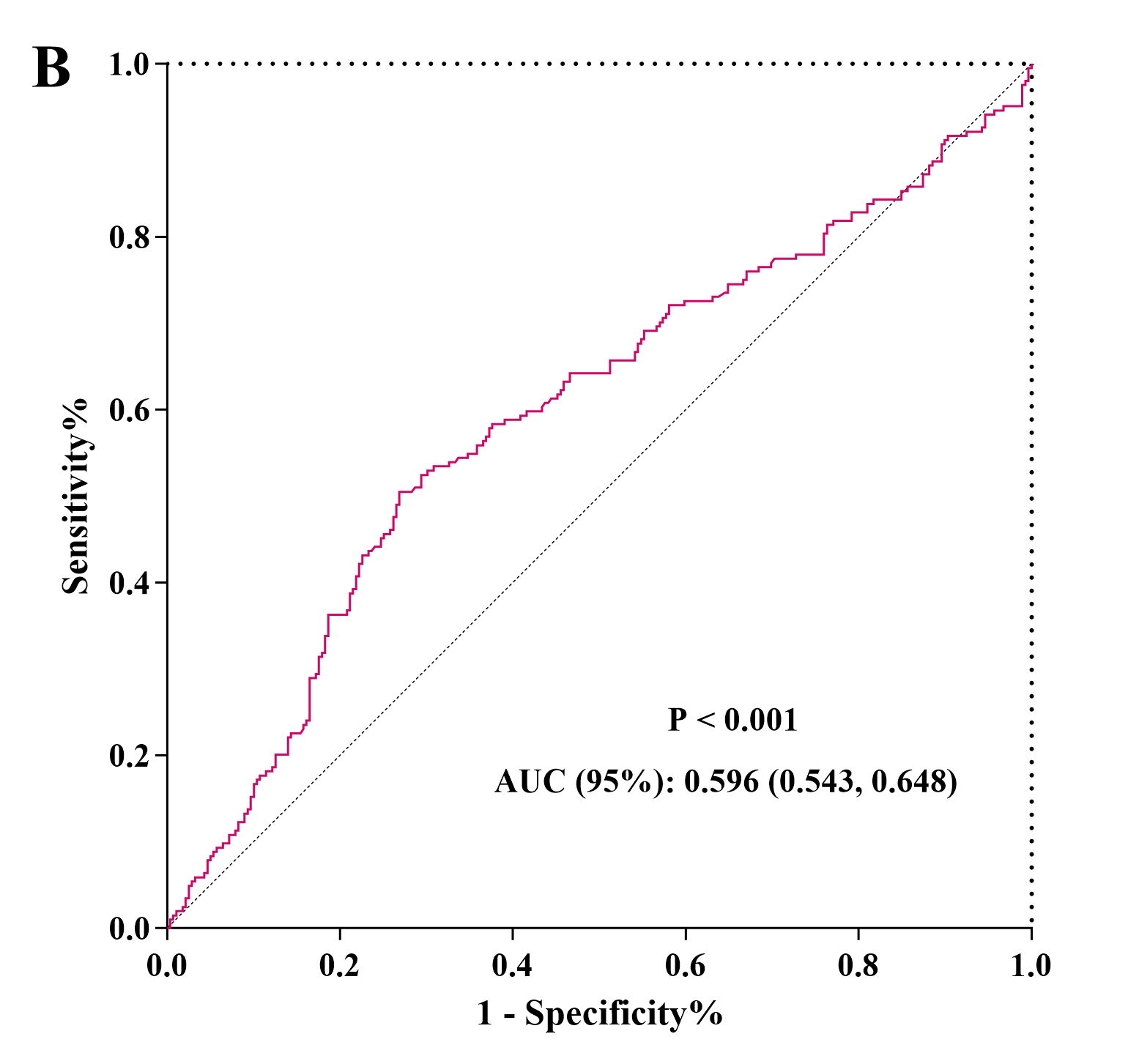


Figure S1. A. The predictive value of SUA for hospital mortality by ROC analysis. B. The predictive value of SUA for ICU mortality by ROC analysis. Abbreviations: SUA, serum uric acid; ICU, Intensive Care Unit; ROC, receiver operating characteristic curve.
