# Supplementary material for: Serum uric acid level as a prognostic biomarker in critically ill patients with sepsis-associated acute kidney injury: A retrospective single-center study
Source: PLoS One. 2025 May 7;20(5):e0321576. doi: 10.1371/journal.pone.0321576 (PMC12057918; doi:10.1371/journal.pone.0321576)
Supplement: S1 Table — (DOCX) [file pone.0321576.s002.docx]

Table S1. Cox proportional hazards regression of the factors influencing all cause mortality of the study population.

| **Variables** | **HR** | **95% CI** | **P-value** |
| --- | --- | --- | --- |
| SUA | 1.001 | 1.000-1.002 | 0.002 |
| HUA | 1.566 | 1.192-2.058 | 0.001 |
| Age | 1.018 | 1.007-1.029 | 0.001 |
| Female | 1.008 | 0.759-1.339 | 0.955 |
| BMI | 0.993 | 0.965-1.022 | 0.635 |
| Smoking | 1.076 | 0.767-1.509 | 0.671 |
| Hypertension | 1.151 | 0.878-1.510 | 0.309 |
| Diabetes | 1.185 | 0.890-1.578 | 0.246 |
| Coronary artery disease | 1.376 | 0.961-1.970 | 0.081 |
| WBC | 1.006 | 0.991-1.022 | 0.411 |
| Neu | 1.008 | 0.992-1.024 | 0.31 |
| Lym | 0.595 | 0.431-0.821 | 0.002 |
| PLT | 0.998 | 0.996-1.000 | 0.015 |
| CRP | 1.001 | 0.999-1.002 | 0.401 |
| Alb | 0.996 | 0.977-1.016 | 0.682 |
| D-dimer | 1.010 | 1.004-1.015 | <0.001 |
| Lactate | 1.067 | 1.035-1.100 | <0.001 |
| APACHE II score | 1.041 | 1.025-1.059 | <0.001 |
| SOFA score | 1.061 | 1.027-1.096 | <0.001 |

Abbreviations: SUA, serum uric acid; HUA, hyperuricemia; BMI, body mass index; WBC, white blood cell; Neu, neutrophil; Lym, lymphocyte; PLT, platelet; CRP, C-reactive protein; Alb, albumin; APACHE II, Acute Physiology and Chronic Health Evaluation II; SOFA, Sequential Organ Failure Assessment.
